# Implementation of the updated 2015 Commission for Hospital Hygiene and Infection Prevention (KRINKO) recommendations “Prevention and control of catheter-associated urinary tract infections” in the hospitals in Frankfurt/Main, Germany

**DOI:** 10.3205/dgkh000274

**Published:** 2016-06-30

**Authors:** Ursel Heudorf, Miriam Grünewald, Ulla Otto

**Affiliations:** 1 Public Health Department, Infectiology and Hygiene, Frankfurt/Main, Germany

**Keywords:** hygiene, infection prevention, urinary catheter, prevention of urinary tract infections, public health office, Commission for Hospital Hygiene and Infection Prevention

## Abstract

**Aim:** The Commission for Hospital Hygiene and Infection Prevention (KRINKO) updated the recommendations for the prevention of catheter-associated urinary tract infections in 2015. This article will describe the implementation of these recommendations in Frankfurt’s hospitals in autumn, 2015.

**Material and methods**: In two non-ICU wards of each of Frankfurt’s 17 hospitals, inspections were performed using a checklist based on the new KRINKO recommendations. In one large hospital, a total of 5 wards were inspected. The inspections covered the structure and process quality (operating instructions, training, indication, the placement and maintenance of catheters) and the demonstration of the preparation for insertion of a catheter using an empty bed and an imaginary patient, or insertion in a model.

**Results:** Operating instructions were available in all hospital wards; approximately half of the wards regularly performed training sessions. The indications were largely in line with the recommendations of the KRINKO. Alternatives to urinary tract catheters were available and were used more often than the urinary tract catheters themselves (15.9% vs. 13.5%). In accordance with the recommendations, catheters were placed without antibiotic prophylaxis or the instillation of antiseptic or antimicrobial substances or catheter flushing solutions. The demonstration of catheter placement was conscientiously performed. Need for improvement was seen in the daily documentation and the regular verification of continuing indication for a urinary catheter, as well as the omission of regular catheter change.

**Conclusion:** Overall, the recommendations of the KRINKO on the prevention of catheter-associated urinary tract infections were adequately implemented. However, it cannot be ruled out that in situations with time pressure and staff shortage, the handling of urinary tract catheters may be of lower quality than that observed during the inspections, when catheter insertion was done by two nurses. Against this background, a sufficient number of qualified staff and regular ward rounds by the hygiene staff appear recommendable.

## Zusammenfassung

**Ziel:** Zur Prävention von Katheter-assoziierten Harnwegsinfektionen hat die Kommission für Krankenhaushygiene und Infektionsprävention (KRINKO) im Jahr 2015 neue Empfehlungen publiziert. In diesem Beitrag wird über die Umsetzung dieser Empfehlungen in Frankfurter Krankenhäusern im Herbst 2015 berichtet.

**Material und Methode:** In jedem der 17 Krankenhäuser der Stadt fanden auf jeweils zwei peripheren Stationen (keine Intensivstationen) anhand einer auf Grundlage der neuen KRINKO-Empfehlung erarbeiteten Checkliste Überprüfungen statt, in einer großen Klinik wurden insgesamt 5 Stationen überprüft. Die Überprüfung umfasste die Struktur- und Prozessqualität (Arbeitsanweisungen, Schulungen, Indikation, das Legen und die Pflege von Kathetern) und die Demonstration des Legens eines Katheters an einem fiktiven Patienten oder einem Modell.

**Ergebnisse:** Alle Stationen verfügten über einschlägige Arbeitsanweisungen, in etwa der Hälfte der Stationen fanden auch regelmäßige Schulungen statt. Die Indikationen entsprachen weitgehend den Empfehlungen der KRINKO. Alternativen zum Harnwegskatheter (HWK) waren vorhanden und wurden häufiger eingesetzt als Harnwegskatheter selbst (15,9% vs. 13,5%). Auf eine Antibiotika-Prophylaxe beim Legen des Katheters, die Instillationen antiseptischer oder antimikrobieller Substanzen oder Spülungen wurde in Übereinstimmung mit den Empfehlungen verzichtet. Auch die Demonstration des Katheter-Legens war fachgerecht. Verbesserungsbedarf wurde bei der Dokumentation der täglichen Überprüfung des Fortbestehens der Indikation für den HWK sowie bei dem „regelmäßigen“ Katheterwechsel gesehen.

**Schlussfolgerung:** Insgesamt wurde eine gute Umsetzung der Empfehlungen der KRINKO zur Prävention von Katheter-assoziierten Harnwegsinfektionen vorgefunden. Es ist aber nicht auszuschließen, dass in Situationen mit Zeitdruck und bei Personalknappheit der Umgang mit Harnwegskathetern weniger positiv verläuft, als wie es im Rahmen der Begehung beobachtet wurde. Vor diesem Hintergrund erscheinen – neben ausreichendem Fachpersonal auf den Stationen – regelmäßige Visiten des Hygienefachpersonals auf den Stationen empfehlenswert.

## Introduction

Urinary tract infections are among the most common nosocomial infections in hospitals, making up 23.2% of the total, together with surgical wound infections (24.3%) and lower respiratory tract infections (21.7%) [1]. Approximately 80% of the nosocomial urinary tract infections are associated with a urinary tract catheter [2]. In 1999, the German Commission for Hospital Hygiene and Infection Prevention (KRINKO) published recommendations for the prevention and control of catheter-associated urinary tract infections [3]. This recommendation has since been revised based on newer literature, and was published in June, 2015 [4].

In long-term care facilities for the elderly, urinary tract infections are also one of the most frequent nosocomial infections. Here, 5% to 12% of the residents have urinary tract catheters, and urinary tract infections are particularly observed in patients with indwelling urinary catheters. For this reason, the KRINKO included comprehensive recommendations for the handling of urinary catheters, for alternatives to indwelling urethral catheters, and for the prevention of urinary tract infections in the publication “Infection prevention in long-term care facilities” [5].

In the following, the implementation of the KRINKO recommendations for the prevention of catheter-associated urinary tract infections in hospitals in Frankfurt/Main in 2015 is presented. In a separate article, the implementation of the KRINKO recommendations for long-term care facilities will be described [6].

## Materials and methods

In the autumn of 2015, employees of the public health authority visited all of Frankfurt’s 17 hospitals, inspecting 37 general wards using a checklist based on the new KRINKO recommendations in order to evaluate their implementation in practice. In each hospital, the inspections were performed on 2 general wards (intensive care units were excluded), but in one large clinic, a total of 5 wards were inspected. Questions referred to the existence of standard operating procedures (SOPs) for the insertion and maintenance of indwelling urinary catheters, training sessions conducted for the correct technique of aseptic catheter insertion, ongoing management of catheters and the recognition of catheter-associated complications, the availability of alternatives to indwelling urinary catheters and the correct handling of the catheter. Furthermore, it was asked who assessed the patient for appropriate indications, which indications prompted a catheterization, who then performed the catheterization, how indications for continued use were assessed and documented, the number of patients on the ward on the day of the survey, how many of these had a urinary catheter and how many had been supplied with alternatives to a catheter.

**Figure 1:**
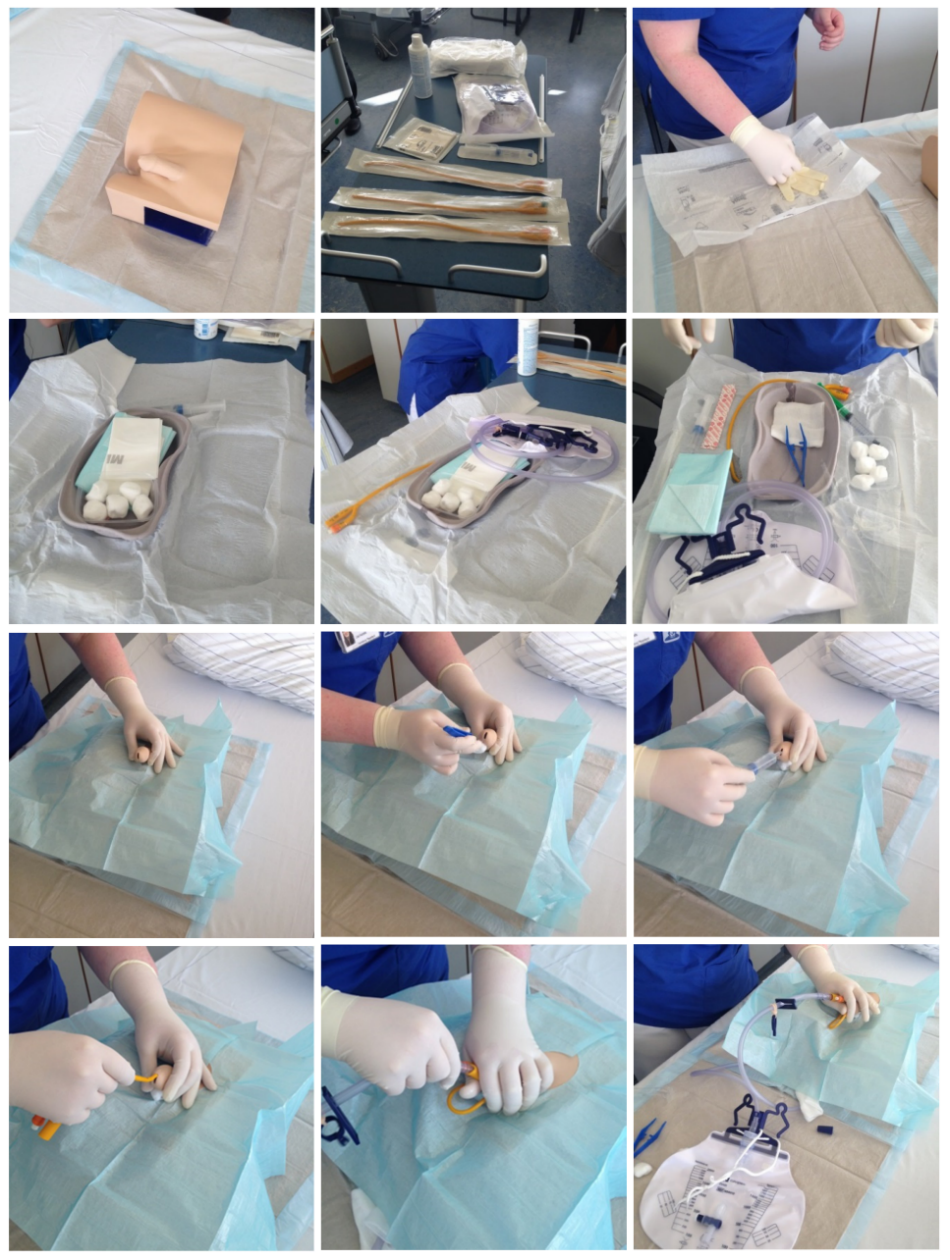
Demonstration of the insertion of a catheter using a patient dummy

Moreover, the public health office staff asked the nursing staff to demonstrate the placement of a urinary tract catheter. Particular attention was paid to the correct preparation of the required materials, the covering of the (fictitious) patient, the disinfection measures (surfaces, hands, mucosa), the insertion and blocking of the catheter. A training manikin was used when available (Figure 1 (Fig. 1)). 

## Results

On 34 of 37 (92%) wards, updated SOPs for the handling of urinary catheters were available. On the remaining 3 wards, the SOPs were in revision after publication of the new recommendation. The performance of regular training on the correct placement of urinary catheters was reported by 46% of the wards. On 78% of the wards, catheters were inserted by the nursing staff, and on less than half of the wards they were also (rarely) placed by physicians (Table 1 (Tab. 1)).

On all wards, the indication for the insertion of a catheter was assessed by a physician; on more than half of the wards, this was done in consultation with the nursing staff. Reported indications for indwelling urethral catheter use were acute urinary retention or monitoring of urine output on 33 (89%) wards, 29 (78%) of the wards also reported support of wound healing on the external genitalia as an indication, 19 (51%) of the wards named prolonged duration of surgery with anticipated large volume infusions, 15 (41%) palliative care, and 6 (16%) urologic surgery as an indication. On 36 (97%) of the wards, the indication was reassessed on a daily basis; however, this was only documented on a third of the wards (Table 1 (Tab. 1)).

As an indication for a catheter change, all wards reported obstruction/technical defects, 35 wards reported catheter-associated urinary tract infections and encrustations, 14 wards reported routinely changing catheters according to the manufacturer’s recommendations, and 2 wards reported changing catheters when patients were referred from a long-term care facility (Table 1 (Tab. 1)).

Alternatives to indwelling urinary catheters were available on all wards. These were mostly incontinence pads and diapers, on 27 wards also pants, and on 21 wards condom catheters (Table 1 (Tab. 1)).

None of the wards administerd prophylactic antibiotics when placing a catheter, used antiseptic or antimicrobial instillations or acidified the urine as infection prophylaxis, or routinely performed bladder irrigations. Both silicon (33 wards) and latex catheters (18 wards) were used, with some wards using both types. Suprapubic catheters are used very rarely for restricted indications (Table 1 (Tab. 1)).

The handling of the closed drainage systems was proficient throughout all hospitals. The staff took care not to kink the catheter tube, and were careful to position the drainage bag below the bladder and made sure that reflux was impossible. In some instances, inventive gadgets and transportation bags had been developed in order to ensure that the drainage bag was safely stored below the bladder but without contact to the floor. However, on 4 wards, contact of the drainage bag with the floor was occasionally observed (Table 2 (Tab. 2)). 

On all wards, the insertion of a catheter was simulated. Three hospitals provided training manikins for this purpose. All nursing staff succeeded in simulating the situation well. The required materials were set up either on a trolley or on the bedside table of the patient. All staff correctly performed the necessary disinfection measures: surface disinfection (bedside table or trolley), then hand disinfection, then disinfection of mucosa. Sterile gloves, cloths, and materials were handled correctly according to the SOPs. Sometimes gowns or aprons were put on (to avoid contamination of the working garments). A few nurses and a urologist were, however, convinced that it is not necessary to wear a dedicated gown or apron during catheterization. The nurses reported that although they could place a catheter on their own (at least if the patient was cooperative), it was easier with a second person to hand them sterile materials; thus, this procedure was chosen for the demonstration. In all cases, the positioning of the drainage bag was checked, either on the bed, the wheelchair, the walker, the chair, or during washing. The nursing staff always kept an eye on correctly positioning the drainage bag below bladder level.

**Figure 2:**
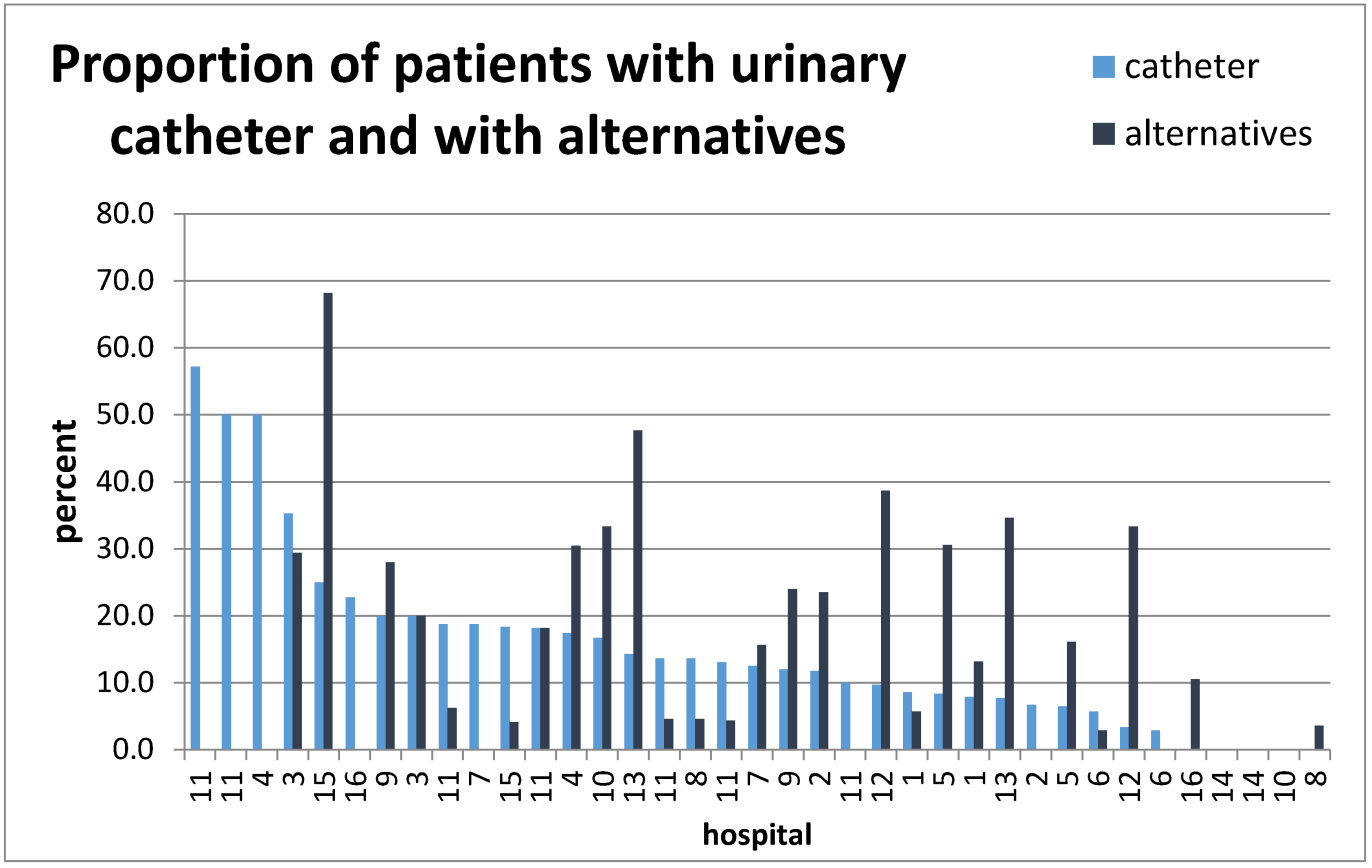
Proportion of patients with urinary catheter (13.5%) and alternatives to urinary catheters (15.9%) on the inspected 37 wards (N=934 patients)

On the days of the inspections, a total of 934 patients were treated on the visited wards. 126 patients (13.5%) had a urinary catheter, 149 (15.9%) were supplied with alternatives to a urinary catheter (diapers, condoms, pants or pads). Large differences were observed between the wards (Figure 2 (Fig. 2)). The highest prevalences for urinary catheters were on an IMC ward (57%) and on urologic wards (2×50% and 1×35%), while the lowest prevalences were found on orthopedic wards (0–9%). On the internal medicine wards, 3–22% of the patients had a urinary catheter, and on the different surgical wards 0–20% did. Three wards had geriatric patients only. On these 0.7 and 14.3% of the patients had a urinary catheter. 

For the 126 patients with a urinary catheter, 153 indications for the catheter were given: most frequently, the monitoring of urine output and urological/neurological diseases and operations (31% respectively), other surgical interventions were reported in 31 patients, and for a further 10 (6.5%), inflammation of the external genitalia was given as the indication. For 11 (7%) patients, other reasons were given, including the wish of the patient (4), catheter already in place upon admission (3), one patient with a persistent vegetative state (PVS), one with incontinence, and two with feverish urinary tract infections (Table 3 (Tab. 3)).

## Discussion

Considering the high proportion of urinary tract infections (23%) of all nosocomial infections in hospitals and the fact that 80% of the urinary tract infections are associated with an indwelling urinary catheter, the prevention of catheter-associated urinary tract infections in hospitals is an important task. It is assumed that up to 70% of all catheter-associated urinary tract infections can be avoided by appropriate hygiene measures, preferably by a combination of measures [7], [8]. In hospitals, urinary tract catheters are placed predominantly in patients on intensive care units. According to data of the German nosocomial infection surveillance system (KISS, module ICU-KISS), the application rate of urinary tract catheters exceeds 80% on ICUs, with an infection rate of 0.93 per 1,000 urinary catheter days [9].

On general wards as well, patients are often supplied with urinary tract catheters. There, the rate of infection is significantly higher with 3.79 per 1,000 urinary catheter days [9]. Possible explanations for this phenomenon could be that on general wards, urinary tract catheters are placed only rarely, the staff is less experienced and possibly less well trained, the handling of urinary catheters for patients who are mobile is more challenging, or that urinary tract infections arise more easily in a setting with a lower antibiotic consumption compared to intensive care units.

Bearing this in mind, the handling of urinary catheters on general wards was inspected in all of Frankfurt’s hospitals on the basis of the KRINKO recommendations for the prevention and control of catheter-associated urinary tract infections, updated in 2015. The inspection included testing the structures (SOPs, training, material) and process quality. Outcomes were not assessed, since a standardized surveillance of catheter-associated urinary tract infections is performed in many hospitals on intensive care units but not on general wards. The evaluation of the structural quality encompassed the availability of SOPs, the performance of training sessions, the assessment and review of appropriate indications, regulations for catheter change, antibiotic prophylaxis and irrigation, and the availability of alternatives to indwelling urinary catheters. To evaluate procedural quality, the handling of the placed catheter was inspected, and on every ward, preparation for insertion of a urinary catheter was demonstrated in an imaginary scenario using an empty bed and including every step of the procedure and required materials, but without involving a patient.

Standard operating procedures were available on all wards or were just being revised due to the recent update of the KRINKO recommendations only a few weeks earlier. However, only around half of the wards reported regularly performing training on the correct insertion and handling of catheters. Nevertheless, all wards performed well during the demonstration of the insertion of a catheter. The individual steps including the necessary surface, hand and mucosa disinfection were demonstrated very well. It must be mentioned, however, that the demonstrations were always performed by two persons (the second to hand the sterile materials to the first). During night shifts or during staff shortages, catheters will often be placed by a nurse working alone, which will place increased demands on the adequate preparation and the systematic workflow, and ultimately may lead to a higher risk of infection.

Three hospitals provided manikins for training purposes. One hospital reported having considerably reduced the rate of urinary tract infections to a very low rate by training with manikins in the ICU-KISS systems.

On all wards, indications for the placement of a urinary catheter were assessed by a physician, usually in consultation with the nursing staff. The indications were manifold, but most frequently, acute urinary retention or monitoring of urine output were reported, as well as support of wound healing around the external genitalia. Only one ward also named urinary incontinence as an indication. According to the KRINKO recommendations, this criterion alone is not an appropriate indication; other incontinence materials should be preferred. Almost all wards reported that the indication was re-assessed daily; however, this was only documented on 30% of the wards, clearly pointing to room for improvement.

Alternative incontinence materials were available on all wards and they were used more often than the urinary catheters: on the respective day of inspection, 15.9% of the patients were supplied with alternatives, while 13.5% of the patients had an indwelling urinary tract catheter. The nursing staff reported that fewer catheters had been placed in recent years because the care concept had changed and physicians were more aware of the associated risk of a urinary tract infection. Additionally, the material of the alternatives had become significantly better and the large diapers and pants can nowadays hold up to 2 liters. For bedridden patients, diapers were mainly used, while mobile patients received pants. Condom catheters were available on all wards, but were only rarely applied.

Catheters were mostly placed by the nursing staff. For complicated cases in which the insertion caused problems, usually an experienced physician was consulted, most often a consulting urologist.

All wards reported changing catheters in case of obstruction or technical defects. 95% of the wards also named encrustations and catheter-associated infections as indications. Over a third of the wards reported regularly changing the catheter according to the respective manufacturer’s recommendations. Upon closer examination, however, these presumed recommendations often did not exist. The proposal was made to get in touch with the other manufacturers to convince them to revise their product instructions in accordance with the new KRINKO recommendations, which state that evidence does not support routine catheter changes for the prevention of infections.

Predominantly, and particularly in cases with a short application time, the economical latex catheters were used.

In accordance with the KRINKO recommendations, antibiotic prophylaxis was not performed and instillations of antiseptic or antibiotic substances were not used. Only one ward reported that before removing a catheter, “bladder training” was carried out, which is not recommended by the KRINKO.

The everyday management of catheters was assessed by questioning and visual inspection. Throughout the wards, the management appeared to be very good.

## Conclusion

The management and handling of urinary/tract catheters in Frankfurt’s hospitals, which was assessed on at least 2 general wards per hospital, was generally appropriate. The indications are largely in accordance with the KRINKO recommendations. Alternatives to urinary tract catheters were available on all wards and were even used more frequently than indwelling urinary catheters. In agreement with the KRINKO recommendations, no antibiotic prophylaxis was performed when inserting the catheter, nor were antiseptic or antimicrobial substances used for bladder irrigation. Room for improvement was seen in the documentation of the daily re-assessment of the indication for a urinary catheter and the “regular” catheter changes.

Good structural quality (SOPs, training, material) is a necessary but not sufficient prerequisite for a good process and outcome quality. A limitation of this survey may be that many items were asked orally; it must also be pointed out that the observation of processes is always subject to an observer bias. Therefore, it cannot be ruled out that in situations in which the staff works under time pressure or shortage of staff, catheters may not be handled as well as when observed during the inspections. Thus, regular visits of the internal hygiene staff on the wards and sufficient staffing of the wards are important aspects for achieving successful prevention of catheter-associated urinary tract infections also in a “normal” situation. Here, the KRINKO gives precise recommendations which translate well into action.

## Notes

### Competing interests

The authors declare that they have no competing interests.

## Figures and Tables

**Table 1:**
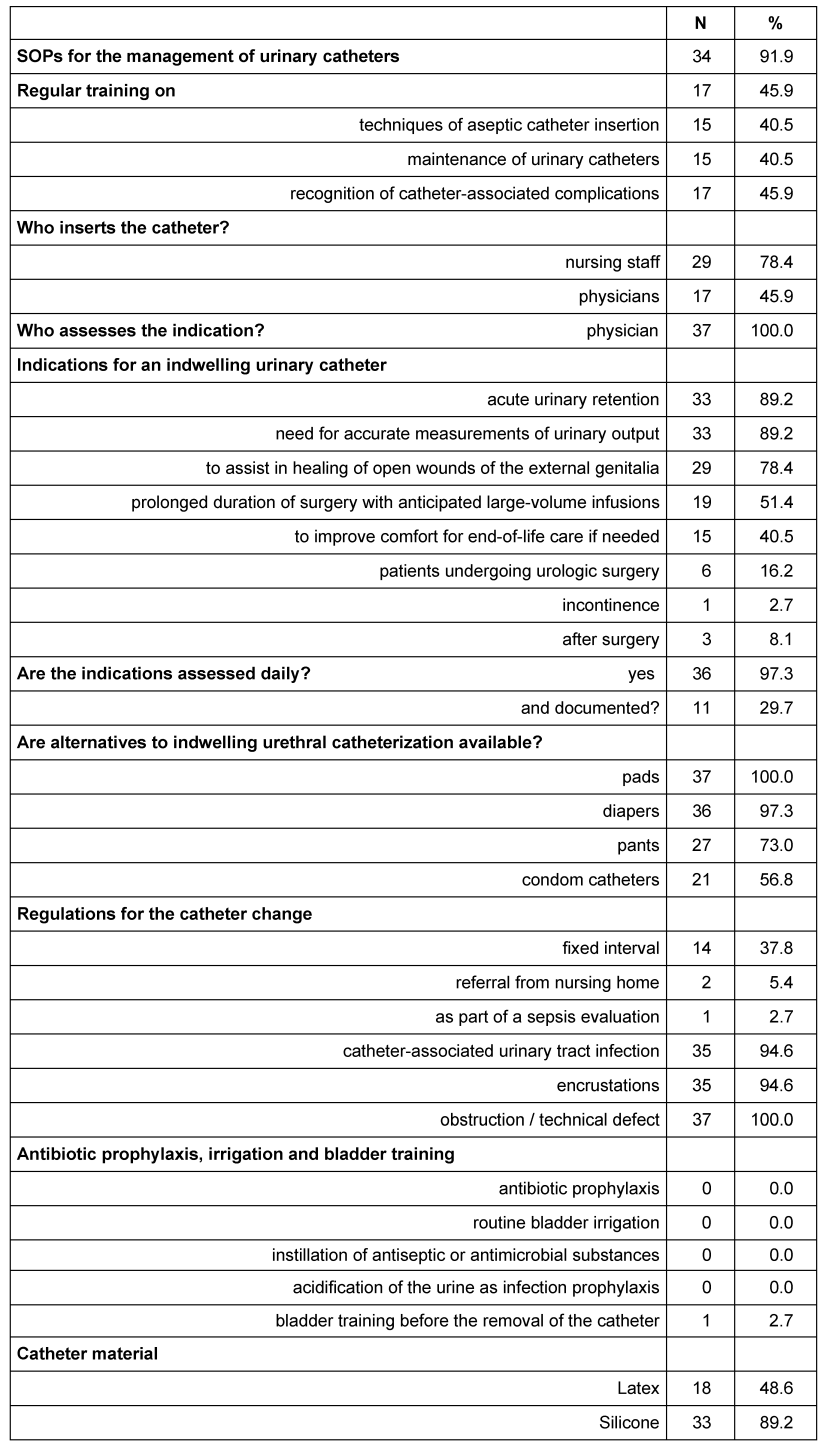
Organizational principles of urinary catheter insertion and maintenance on 37 wards in hospitals in Frankfurt/Main, 2015

**Table 2:**
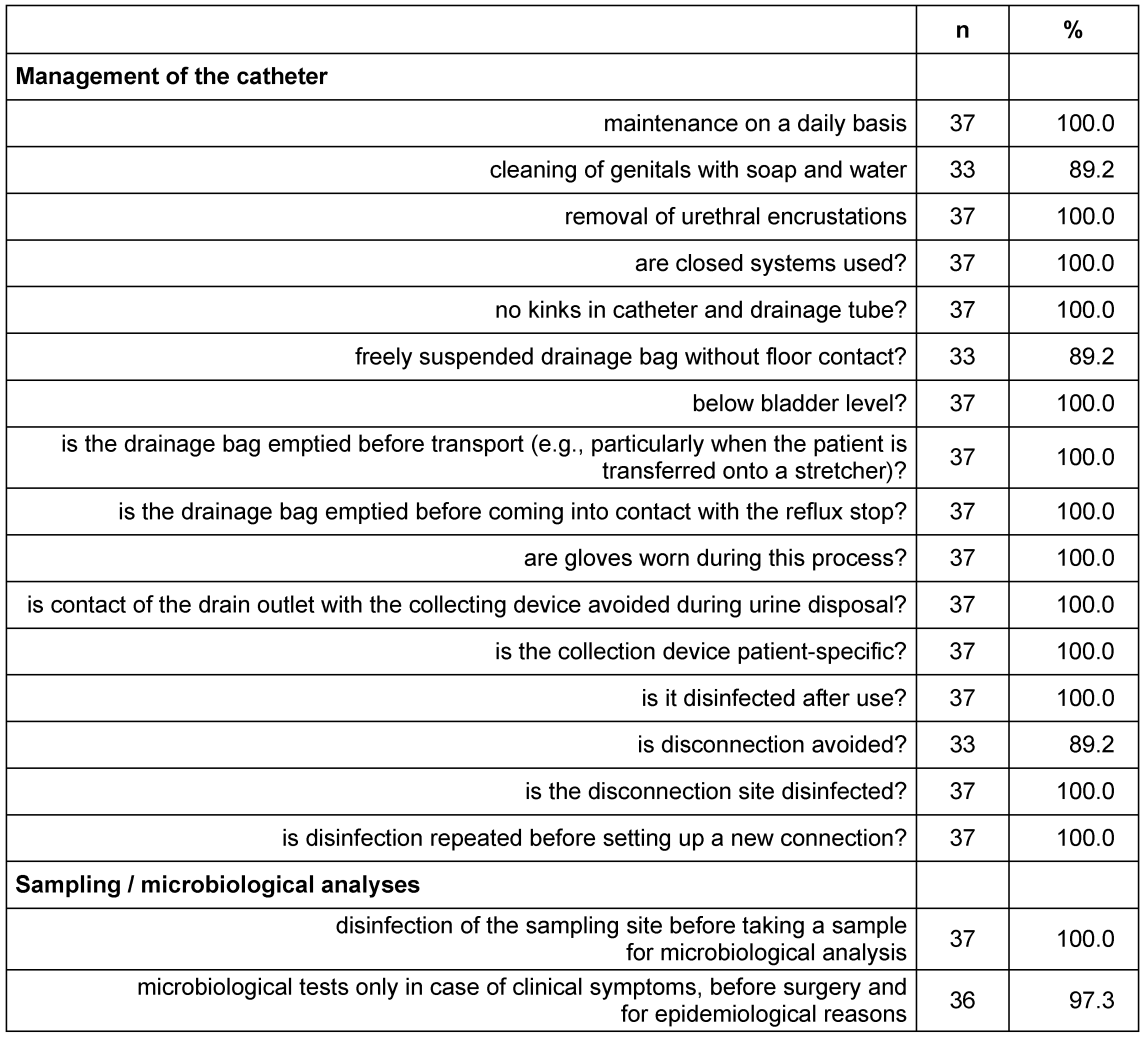
Management of urinary catheters on 37 wards in 17 hospitals in Frankfurt/Main, 2015

**Table 3:**
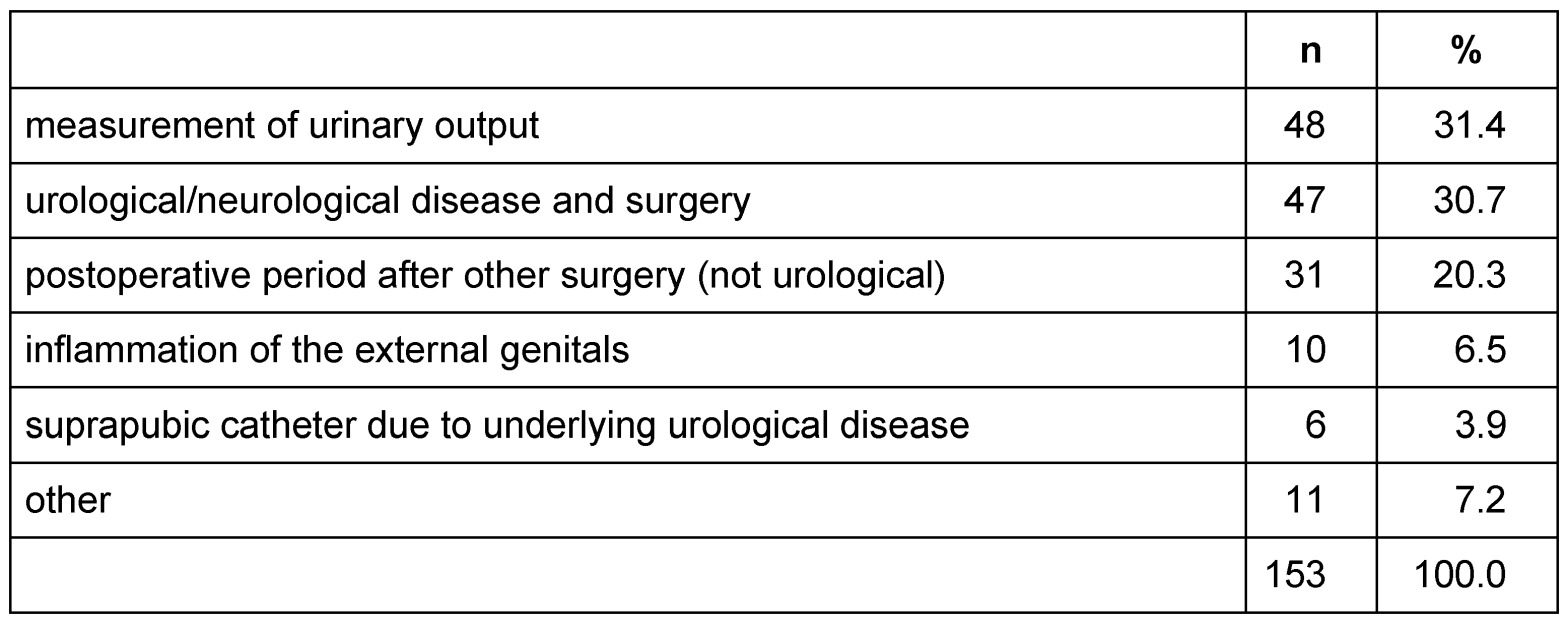
Indications for the usage of a urinary catheter in hospitals (153 indications for 126 patients)
